# Supplementary material for: Deciphering the evolution of composite-type GSKIP in mitochondria and Wnt signaling pathways
Source: PLoS One. 2022 Jan 20;17(1):e0262138. doi: 10.1371/journal.pone.0262138 (PMC8775565; doi:10.1371/journal.pone.0262138)
Supplement: S1 Table — T: type, G: group. (DOCX) [file pone.0262138.s001.docx]

**Supplement table 1: Quote terms: SSF103107, PF05303, IPR007967 and IPR023231 of all species in Uniprot search.**

| **NO** | **Species** | **Domain** |
| --- | --- | --- |
| 1 | Caenorhabditis elegans | **IPR007967(G1)** |
|  |  | **PF05303 (T1)** |
|  |  | **SSF103107(T3)** |
| 2 | Dictyostelium discoideum (Slime mold) | **IPR007967(G1)** |
|  |  | **PF05303 (T1)** |
|  |  | **SSF103107(T3)** |
| 3 | Saccharomyces cerevisiae (strain ATCC 204508/S288c) (Baker's yeast) | **SSF103107(T3)** |
| 4 | Schizosaccharomyces pombe (strain 972/ ATCC 24843) (Fission yeast) | **SSF103107(T3)** |
| 5 | Candida albicans (strain SC5314/ ATCC MYA-2876) (Yeast) | **SSF103107(T3)** |
| 6 | Lodderomyces elongisporus (strain ATCC 11503/ CBS 2605/ JCM 1781/ NBRC 1676 / NRRL YB-4239)(Yeast) | **SSF103107(T3)** |
| 7 | Schistosoma haematobium (Blood fluke) | **IPR007967(G1)** |
|  |  | **PF05303 (T1)** |
|  |  | **SSF103107(T3)** |
| 8 | Lingula unguis | **IPR007967(G1)** |
|  |  | **PF05303 (T1)** |
|  |  | **IPR023231(G3)** |
|  |  | **SSF103107(T3)** |
| 9 | Drosophila melanogaster (Fruit fly) | **IPR007967(G1)** |
|  |  | **PF05303 (T1)** |
|  |  | **IPR023231(G3)** |
|  |  | **SSF103107(T3)** |
| 10 | Culex quinquefasciatus (Southern house mosquito) (Culex pungens) | **IPR007967(G1)** |
|  |  | **PF05303 (T1)** |
|  |  | **SSF103107(T3)** |
| 11 | Aedes aegypti (Yellowfever mosquito) (Culex aegypti) | **IPR007967(G1)** |
|  |  | **PF05303 (T1)** |
|  |  | **SSF103107(T3)** |
| 12 | Aedes albopictus (Asian tiger mosquito) (Stegomyia albopicta) | **IPR007967(G1)** |
|  |  | **PF05303 (T1)** |
|  |  | **SSF103107(T3)** |
| 13 | Brabchiostoma floridae (Florida lancelet) (Amphioxus) | **SSF103107(T3)** |
| 14 | Danio rerio (Zebrfish) (Brachydanio rerio) | **SSF103107(T3)** |
| 15 | Homo sapiens (Human) | **SSF103107(T3)** |
| 16 | Rattus norvegicus (Rat) | **SSF103107(T3)** |
| 17 | Mus musculus (Mouse) | **SSF103107(T3)** |
| 18 | Gallus gallus (Chicken) | **SSF103107(T3)** |
| 19 | Desulfuribacillus alkaliarsenatis | **IPR007967(G1)** |
|  |  | **PF05303 (T1)** |
