# Supplementary material for: Deciphering the evolution of composite-type GSKIP in mitochondria and Wnt signaling pathways
Source: PLoS One. 2022 Jan 20;17(1):e0262138. doi: 10.1371/journal.pone.0262138 (PMC8775565; doi:10.1371/journal.pone.0262138)
Supplement: S2 Table — (DOCX) [file pone.0262138.s002.docx]

| Entry | Entry name | Status | Protein names | Gene names | Organism | Length |
| --- | --- | --- | --- | --- | --- | --- |
| A0A671EDT8 | A0A671EDT8_RHIFE | unreviewed | CLUH | CLUH mRhiFer1_003033 | Rhinolophus ferrumequinum (Greater horseshoe bat) | 1298 |
| A0A671E8W4 | A0A671E8W4_RHIFE | unreviewed | CLUH | CLUH mRhiFer1_003033 | Rhinolophus ferrumequinum (Greater horseshoe bat) | 1349 |
| A0A6J2M6I5 | A0A6J2M6I5_9CHIR | unreviewed | CLUH | CLUH | Phyllostomus discolor (pale spear-nosed bat) | 1349 |
| A0A6J2M627 | A0A6J2M627_9CHIR | unreviewed | CLUH | CLUH | Phyllostomus discolor (pale spear-nosed bat) | 1348 |
| S7P578 | S7P578_MYOBR | unreviewed | CLUH | D623_10015695 | Myotis brandtii (Brandt's bat) | 1381 |
| K9IP54 | K9IP54_DESRO | unreviewed | CLUH |  | Desmodus rotundus (Vampire bat) | 1311 |
| A0A7E6EDX8 | A0A7E6EDX8_9CHIR | unreviewed | CLUH | CLUH | Phyllostomus discolor (pale spear-nosed bat) | 1355 |
| L5JTY7 | L5JTY7_PTEAL | unreviewed | CLUH | PAL_GLEAN10020025 | Pteropus alecto (Black flying fox) | 1309 |
| L5LKR6 | L5LKR6_MYODS | unreviewed | CLUH | MDA_GLEAN10016906 | Myotis davidii (David's myotis) | 1353 |
| A0A7J8CWU3 | A0A7J8CWU3_MOLMO | unreviewed | Clustered mitochondria-like protein | HJG59_003027 | Molossus molossus (Pallas' mastiff bat) (Vespertilio molossus) | 1349 |
| A0A7J7T3W7 | A0A7J7T3W7_MYOMY | unreviewed | Clustered mitochondria-like protein | mMyoMyo1_003019 | Myotis myotis (Greater mouse-eared bat) | 132 |
| A0A7J8G626 | A0A7J8G626_ROUAE | unreviewed | Clustered mitochondria-like protein | HJG63_003028 | Rousettus aegyptiacus (Egyptian rousette) (Egyptian fruit bat) | 691 |
| A0A7J7T4E4 | A0A7J7T4E4_MYOMY | unreviewed | Clustered mitochondria-like protein | mMyoMyo1_003019 | Myotis myotis (Greater mouse-eared bat) | 380 |
| A0A7J8G7J3 | A0A7J8G7J3_ROUAE | unreviewed | Clustered mitochondria-like protein | HJG63_003028 | Rousettus aegyptiacus (Egyptian rousette) (Egyptian fruit bat) | 1298 |
| A0A7J7T4N0 | A0A7J7T4N0_MYOMY | unreviewed | Clustered mitochondria-like protein | mMyoMyo1_003019 | Myotis myotis (Greater mouse-eared bat) | 1347 |
| A0A7J8G610 | A0A7J8G610_ROUAE | unreviewed | Clustered mitochondria-like protein | HJG63_003028 | Rousettus aegyptiacus (Egyptian rousette) (Egyptian fruit bat) | 1349 |
| A0A7J8G6W5 | A0A7J8G6W5_ROUAE | unreviewed | Clustered mitochondria-like protein | HJG63_003028 | Rousettus aegyptiacus (Egyptian rousette) (Egyptian fruit bat) | 1094 |
| A0A7J7T4K5 | A0A7J7T4K5_MYOMY | unreviewed | Clustered mitochondria-like protein | mMyoMyo1_003019 | Myotis myotis (Greater mouse-eared bat) | 541 |
| A0A7J7T3Z6 | A0A7J7T3Z6_MYOMY | unreviewed | Clustered mitochondria-like protein | mMyoMyo1_003019 | Myotis myotis (Greater mouse-eared bat) | 1374 |
| A0A7J8G693 | A0A7J8G693_ROUAE | unreviewed | Clustered mitochondria-like protein | HJG63_003028 | Rousettus aegyptiacus (Egyptian rousette) (Egyptian fruit bat) | 914 |
| A0A7J7T3T7 | A0A7J7T3T7_MYOMY | unreviewed | Clustered mitochondria-like protein | mMyoMyo1_003019 | Myotis myotis (Greater mouse-eared bat) | 1348 |
| A0A7J8G6E7 | A0A7J8G6E7_ROUAE | unreviewed | Clustered mitochondria-like protein | HJG63_003028 | Rousettus aegyptiacus (Egyptian rousette) (Egyptian fruit bat) | 1348 |
| A0A7J7TD17 | A0A7J7TD17_RHIFE | unreviewed | Clustered mitochondria-like protein | mRhiFer1_003033 | Rhinolophus ferrumequinum (Greater horseshoe bat) | 1348 |
| A0A7J7SME3 | A0A7J7SME3_PIPKU | unreviewed | Clustered mitochondria-like protein | mPipKuh1_002980 | Pipistrellus kuhlii (Kuhl's pipistrelle) | 1348 |
| A0A7J7SMM3 | A0A7J7SMM3_PIPKU | unreviewed | Clustered mitochondria-like protein | mPipKuh1_002980 | Pipistrellus kuhlii (Kuhl's pipistrelle) | 1349 |
| A0A7J7SMB1 | A0A7J7SMB1_PIPKU | unreviewed | Clustered mitochondria-like protein | mPipKuh1_002980 | Pipistrellus kuhlii (Kuhl's pipistrelle) | 1298 |
| A0A7E6EDX8 | A0A7E6EDX8_9CHIR | unreviewed | CLUH | CLUH | Phyllostomus discolor (pale spear-nosed bat) | 1355 |
| L5JTY7 | L5JTY7_PTEAL | unreviewed | CLUH | PAL_GLEAN10020025 | Pteropus alecto (Black flying fox) | 1309 |
