# Supplementary figures and images for: Deciphering the evolution of composite-type GSKIP in mitochondria and Wnt signaling pathways
Source: PLoS One. 2022 Jan 20;17(1):e0262138. doi: 10.1371/journal.pone.0262138 (PMC8775565; doi:10.1371/journal.pone.0262138)

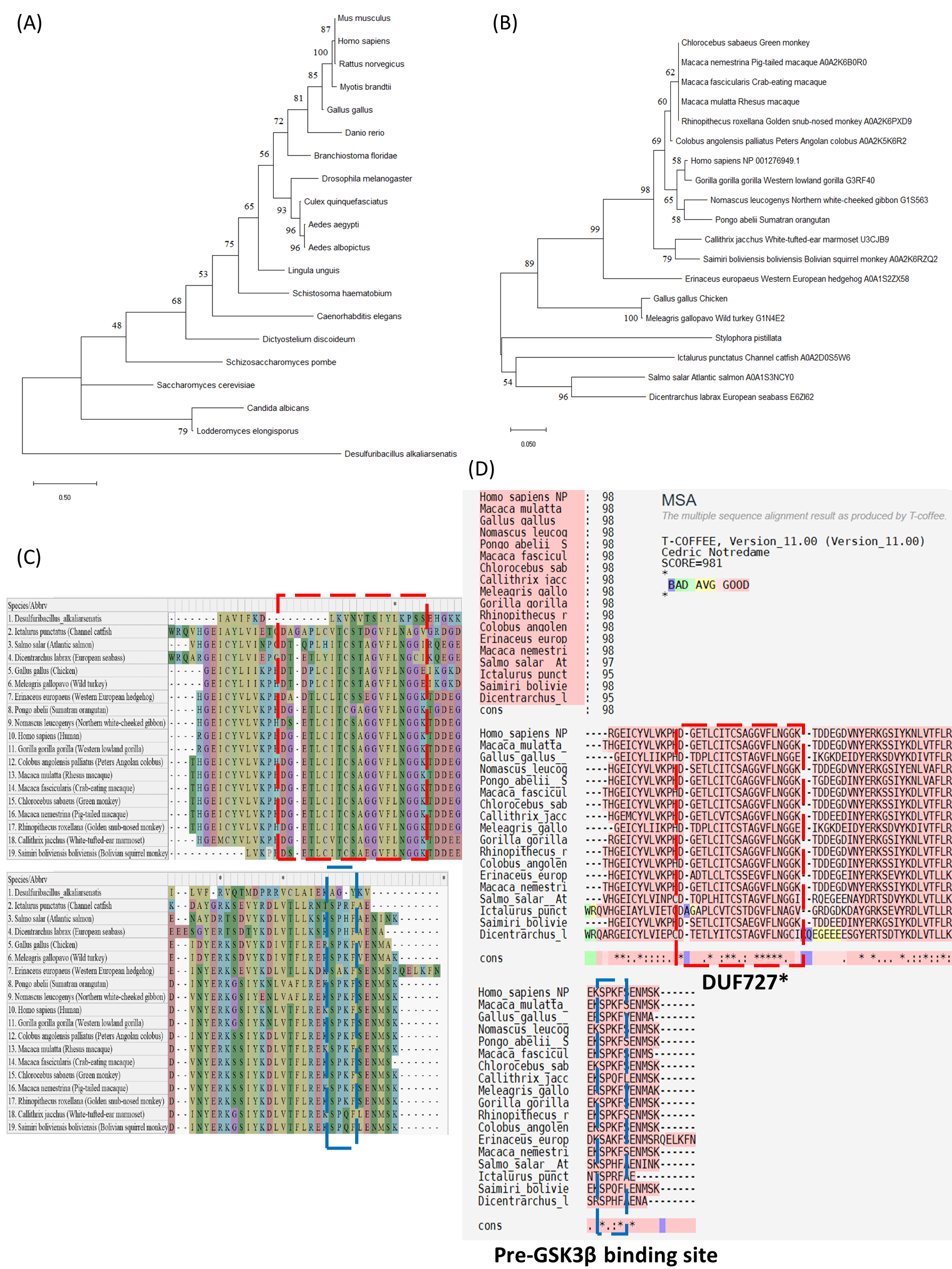

Supplement: S1 Fig — Sequence alignment and phylogenetic analysis of ARMC4 from 18 vertebrate species along with a bacterium are shown. A and B, Phylogenetic tree of CLU1 and ARMC4 generated using MEGA-X, respectively. Boot strapping values represented the likelihood function for each other species as indicated. The boot strapping test measures the internal consistency of data produced above than 0.5 (50%) of the bootstrap replicates are consistent. C, Multiple sequence alignment with respect to the DUF727 region of GSKIP orthologs was conducted using ClustalW. D, T-Coffee estimates of alignment accuracy improved phylogenetic tree reconstruction. The conserved residues are indicated with asterisks, and residues with high similarity among the orthologs are marked with dots at the bottom. The label * indicates the possible region of DUF727 in ARMC4. (TIF) [file pone.0262138.s003.tif]
