# Supplementary material for: Deciphering the evolution of composite-type GSKIP in mitochondria and Wnt signaling pathways
Source: PLoS One. 2022 Jan 20;17(1):e0262138. doi: 10.1371/journal.pone.0262138 (PMC8775565; doi:10.1371/journal.pone.0262138)
Supplement: Attachment — Submitted filename: Response to reviewer.docx [file pone.0262138.s005.docx]

**Response to Reviewers**

Please use the space provided to explain your answers to the questions above. You may also include additional comments for the author, including concerns about dual publication, research ethics, or publication ethics. (Please upload your review as an attachment if it exceeds 20,000 characters)

Reviewer #1: Although the authors have addressed the concerns of this reviewer there are still a few things to clarify.

***Q1. It still has not been properly defined what is meant by simple-type and composite forms of GSKIP. In line, in the introduction the authors list all known functions of GSKIP but do not elaborate on which GSKIP form, simple-type or composite, can be allocated to which function. Please do so in order to put the reader into a position to follow.***

A1. Sorry for the confusion of “simple-type and composite forms of GSKIP”. GSKIP has two subtypes, there is only one simple-type GSKIP, all the functions described in the introduction part belong to this type. However, there are four groups of composite-type GSKIP (clustered mitochondria protein 1 (CLU1) and Mitofilin in mitochondria, heat shock protein 70 (Hsp70), and Armadillo repeat-containing 4 (ARMC4). In this article, we try to decipher and emphasize the origin and evolution of these four groups of composite-type GSKIP (Referring lines 102-109).

Reviewer #2: ***All suggestions and comments were addressed by the authors. I have no further comments or suggestions.***

Thank you for all the suggestions and efforts.

Reviewer #3: Deciphering the evolution of composite-type GSKIP in mitochondria and Wnt signaling pathways A considerable improvement has already taken place in the comprehensibility of the manuscript, however, the manuscript is still difficult to follow and needs to rethink again. Besides, some issues should be addressed.

***Q1. the list of organisms from lines 275 to 287 is referring only to CLU1, but in the tree, there are more species. Maybe the authors would like to refer to Fig S1A instead of Fig 2 in line 274.***

A1. Thank you for your comment. We have referred to figure S1A in line 274.

***Q2. Because the four composite-type GSKIP differ a lot in their domains the bootstraps in figure 2 are not be reliable. A bootstrap support above 95% is very good and very well accepted and a bootstrap support between 75% and 95% is reasonably good, anything below 75% is very poor support and anything below 50% is of no use. If authors want to show phylogenetic relationships between the four composite-type GSKIP I recommend them to use only the orthologous domains to construct the tree.***

A2. We agree. In general, we believe that a reliable bootstrap between 75% and 95% is for individual orthologous domains to construct the tree. Our data is for four groups of orthologous domains (from human to bacteria should be more diverse) to construct the tree, therefore, a bootstrap 50% could be acceptable.

***Q3. Another issue is that the authors claim in line 305 the mutation of Leu130 always causes abolition of the interaction, however in full-length GSKIP the interaction is positive for 3 out of 4 mutations tested.***

A3. Thank you for your comment. Since Leu130 plays a crucial role in GSKIP binding to GSK3beta for simple-type GSKIP, our mutagenesis assay (table 1) of the full length of GSKIP (1-139) and GSKIPtide (115-139) 3 out of 4 mutations due to the conformational change (particularly for Pro130 causes distort helix in both full-length and GSKIPtide). (Please see references 1,3,6 and 20 in this study.)
